# 3D-Printed Fiber-Reinforced Polymer Composites by Fused Deposition Modelling (FDM): Fiber Length and Fiber Implementation Techniques

**DOI:** 10.3390/polym14214659

**Published:** 2022-11-01

**Authors:** Khairul Izwan Ismail, Tze Chuen Yap, Rehan Ahmed

**Affiliations:** 1 School of Engineering and Physical Sciences, Heriot-Watt University Malaysia, No. 1, Jalan Venna P5/2, Precinct 5, Putrajaya 62200, Malaysia; 2 School of Engineering and Physical Sciences, Heriot-Watt University, Edinburgh EH14 4AS, UK

**Keywords:** additive manufacturing, fused filament fabrication, 3D printing, fiber reinforced thermoplastics, fiber reinforced polymer composites, continuous fiber, short fiber

## Abstract

Fused Deposition Modelling (FDM) is an actively growing additive manufacturing (AM) technology due to its ability to produce complex shapes in a short time. AM, also known as 3-dimensional printing (3DP), creates the desired shape by adding material, preferably by layering contoured layers on top of each other. The need for low cost, design flexibility and automated manufacturing processes in industry has triggered the development of FDM. However, the mechanical properties of FDM printed parts are still weaker compared to conventionally manufactured products. Numerous studies and research have already been carried out to improve the mechanical properties of FDM printed parts. Reinforce polymer matrix with fiber is one of the possible solutions. Furthermore, reinforcement can enhance the thermal and electrical properties of FDM printed parts. Various types of fibers and manufacturing methods can be adopted to reinforce the polymer matrix for different desired outcomes. This review emphasizes the fiber types and fiber insertion techniques of FDM 3D printed fiber reinforcement polymer composites. A brief overview of fused deposition modelling, polymer sintering and voids formation during FDM printing is provided, followed by the basis of fiber reinforced polymer composites, type of fibers (synthetic fibers vs. natural fibers, continuous vs. discontinuous fiber) and the composites’ performance. In addition, three different manufacturing methods of fiber reinforced thermoplastics based on the timing and location of embedding the fibers, namely ‘embedding before the printing process (M1)’, ‘embedding in the nozzle (M2)’, and ‘embedding on the component (M3)’, are also briefly reviewed. The performance of the composites produced by three different methods were then discussed.

## 1. Introduction

Additive manufacturing (AM) or 3-dimensional printing (3DP) technology is one of the most promising areas in component manufacturing. AM has paved its way into application areas ranging from automotive [1], construction [2], aerospace [3] and consumer products to biomedical products such as prosthetics [4]. AM refers to a group of fabrication techniques where parts are fabricated layer-by-layer directly from a computer-aided design (CAD) file. AM technology is a very broad term that encompasses many methods such as Stereolithography (SLA) of a photopolymer liquid [5], Laminated Object Manufacturing (LOM) from plastic laminations [6], Selective Laser Sintering (SLS) from plastic or metal powder [7] and Fused Deposition Modelling (FDM) from plastic filaments [8]. Since 1980, many studies have been conducted to maximize the potential applications of these technologies, as AM is well-known and still a far more cost-effective alternative to subtractive manufacturing technologies such as milling, drilling and turning [9]. FDM, also called Fused Filament Fabrication (FFF), is one of the most popular techniques due to its relatively low cost, low material wastage and ease of use. Nowadays, most people can even purchase and use this technique at home. However, FDM 3D print is yet to replace conventional manufacturing in producing functional parts. FDM 3D print parts are weaker than conventionally manufactured counterparts due to their layer-by-layer fabrication method. Research has been carried out to improve the mechanical properties of FDM printed parts by using various methods, such as optimizing printing parameters, annealing, snap-fitting [10], printing in an oxygen free environment [11], mechanical pressing [12] and fiber reinforced thermoplastics. Of all the methods, fiber reinforced polymer composites (FRPC) are known to have high stiffness, strength, damage tolerance, fatigue resistance and corrosion resistance. FRPCs are produced by adding fibers or particles into the thermoplastic matrix to improve the mechanical strength of the printed components [13]. This method reduces voids and increases interlaminar bonding between the deposited filaments. There are two types of fiber reinforcement: continuous and discontinuous, depending on fiber length. Fiber reinforced composites have a long history and are traditionally produced by techniques like hand lay-up, molding, etc. FDM is a relatively new technique for manufacturing fiber reinforced polymer composites. Research in FDM 3D printed fiber reinforced polymer composites has flourished recently. Previous works in FDM 3D printed fiber reinforced polymer composites have been reviewed by several state-of-the-art review papers with a different emphasis [14,15,16,17,18,19,20,21]. Additive manufactured fiber reinforced polymer composites produced by different AM techniques such as FDM, DIW, SLS, SLA, and Laminated Object Manufacturing (LOW) and their mechanical behavior were summarized by Li et al. [14]. The matrix and materials used in fiber reinforced additive manufacturing and the mechanical behavior were reviewed by Fidan et al. [15]. Understanding and optimizing the printing parameters of the FDM process to achieve the desired mechanical properties of the printed part was summarized by Krajangsawasdi et al. [16] and Shanmugam et al. [17]. The FDM printed natural fiber reinforced polymer produced was reviewed by Mazzanti et al. [18], whereas FDM printed discontinuous fiber polymer composites were summarized by Hu et al. [19]. Lastly, the fabrication techniques and application of 3D printed anisotropic polymer materials were presented by Chen et al. [20] and Xu et al. [21]. However, there has not been an in-depth discussion on fabrication techniques, and fiber types of FDM 3D printed fiber reinforced polymer. Therefore, this review aims to summarize recent progress in FDM 3D printed fiber reinforced polymers and emphasize fiber type and fabrication techniques. The following section summarizes the working principle for FDM 3D printing, especially the polymer sintering and forming of voids. Previous attempts to improve print quality by optimizing printing parameters are presented in (Section 3). Since this review’s focus is on fiber as reinforcement, the basis of fiber reinforced polymer composites and the type of fibers (synthetic fibers vs. natural fibers, continuous vs. discontinuous fiber) will be given in Section 4. We highlight the manufacturing methods of fiber reinforced polymer composites in Section 5, and then propose opportunities for future development in Section 6.

## 2. Polymer Sintering and Voids Formation in Fused Deposition Modelling

### 2.1. Fused Deposition Modelling Process

**Figure 1:**
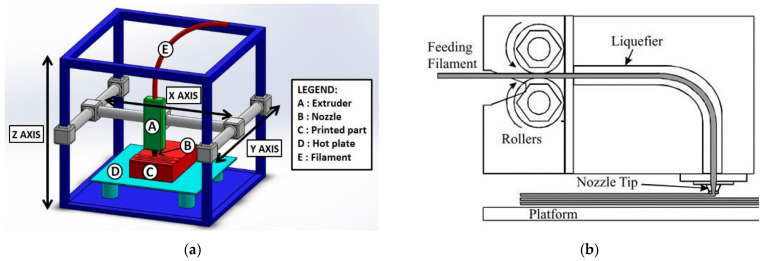
(**a**) Schematic representation of a typical FDM setup, [18] (**b**) schematic of the FDM extrusion head and filament deposition process, reprinted/adapted with permission from [22], 2008, Emerald Publishing, Bingley, UK.

The process of creating an object with an FDM printer begins with the product design using CAD software such as CATIA and SOLIDWORKS, which is saved in a Surface Tessellation Language (STL) file. Before such a file can be printed, it must be converted into a format that the 3D printer can understand, namely a G-code file. Slicer software such as Cura, Ideamaker and Simpliy 3D are used to convert the STL file into a G-code file. The G-code contains commands for moving parts within the printer. It consists of G- and M-commands that have assigned actions and movements in x-, y- and z-directions of the nozzle and bed of the FDM printer. Figure 1 shows a schematic diagram of a typical FDM printer setup and filament deposition process.

A 3D geometry is produced in the FDM process by building up an extruded thermoplastic filament layer-by-layer. The filament is fed through the extrusion head (nozzle), which is heated to a semi-liquid state and applied to the build platform through a nozzle in layers. Each layer is bonded to the adjacent layers in the semi-liquid state. Thus, it is crucial to control the feed rate of the printer to ensure that the previous layer does not solidify too early. Feed rate can be easily adjusted in the slicer software. Figure 1b shows a schematic diagram of the FDM extrusion head and filament deposition process. The filament is first driven into the print head by rollers. As it passes through a liquefier, the feedstock is heated by a heater to a viscous melt and pushed out of the print nozzle by the incoming still-solid filament.

### 2.2. Polymer Sintering of Deposited Thermoplastics

**Figure 2:**
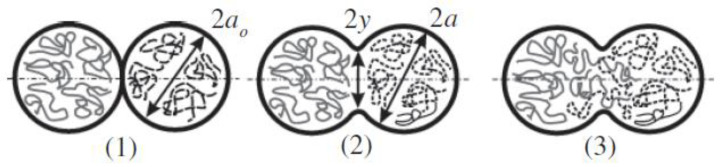
Formation of the neck between filaments: (**1**) surface contacting, (**2**) neck growth, and (**3**) diffusion at interface, Reprinted with permission from [24], 2004, Elsevier, Amsterdam, The Netherlands.

The FDM process uses a heated nozzle to melt and extrude thermoplastic filaments such as Acrylonitrile-butadiene-styrene (ABS), poly-lactic acid (PLA), nylon, polypropylene (PP), polyethylene (PE), and so on. These materials are common thermoplastics used in 3D printing. Each material has a different melting point, and the printer must be set accordingly. An error in setting up the temperature of the feedstock material will affect the cosmetic and strength of 3D printed products. During the FDM process, each filament extruded through the heated nozzle solidifies and forms a cross-bond with the adjacent filaments extruded previously. These filaments form a bridge between them, known as the “neck” by the process of polymer sintering [23]. This bond, which is responsible for growing the necks within a layer, may be termed as the “intra-layer bonding”. Since the temperature of the previously solidified layer is still high, there is a good tendency for similar bonds to form between the filaments of the two successive layers, which can be termed as “inter-layer bonding”. Figure 2 describes the surface contact stage, neck growth and molecular diffusion of the deposited filaments [24].

**Figure 3:**
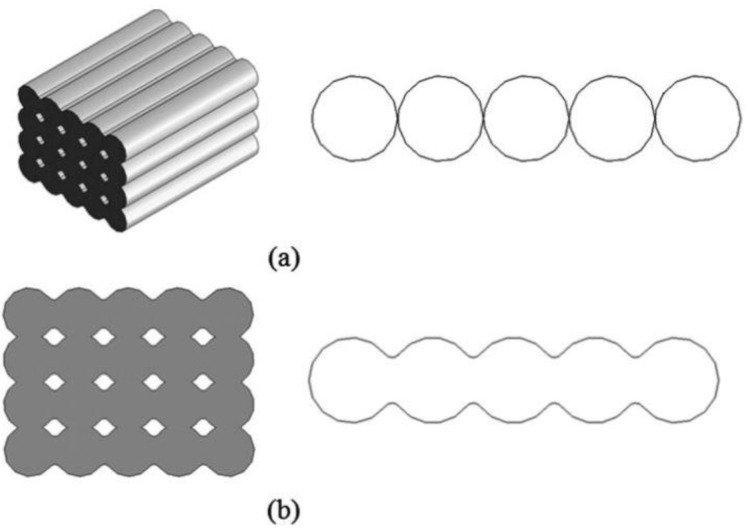
Schematic of: (**a**) assumed position of filaments before any bond form and, (**b**) actual position of filaments after intra-layer and inter-layer bonding is formed. Reprinted with permission from [23], 2014, Taylor and Francis, Abingdon, UK.

**Figure 4:**
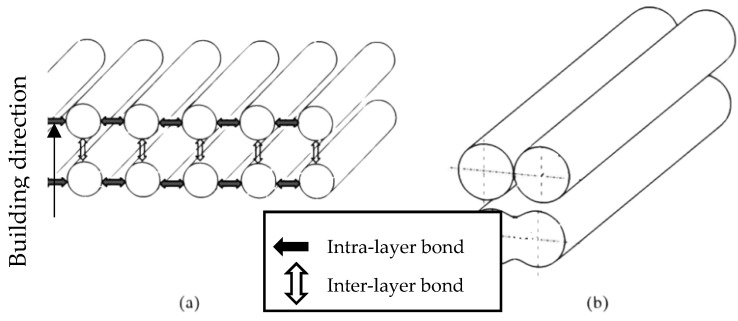
(**a**) Inter-layer and intra-layer bonding in FDM, (**b**) stages of bond formation in FDM. Reprinted with permission from [23], 2014, Taylor and Francis, Abingdon, UK.

Gurrala and Regalla et al. [23] also investigated the effects of inter-layer bonding, intra-layer bonding and neck formation between adjacent filaments on the tensile strength of FDM products, both experimentally and theoretically. They found that in the FDM sample with 0° raster angle, the failure of a specimen was due to inter-layer fracture, whereas at 45° raster angle, the specimens failed due to both inter-layer and intra-layer fracture. This research has shown that inter-layer and intra-layer bonds play an important role in the mechanical properties of FDM products. Figure 3 and Figure 4 are schematic diagrams of multi-layer extruded filaments. The strength of printed parts depends on these two interlaminar bonds. To improve these interlaminar bonds, much research has been done focusing on the printing parameters. They believe that an optimal setup results in high strength FDM products with great interlaminar bonding. An overview of these research will be presented in the next section (Section 3).

Multiple attempts have been made to numerically model the sintering process of polymers based on heat transfer calculations. Early work by Yardimci et al. [25,26] presented different modelling approaches to capture the heat transfer between printed beads, but did not consider the polymer flow dynamics. Bellehumeur et al. [24] used a model based on a polymer sintering model described by Pokluda et al. [27]. Pokluda et al. performed an energy balance between surface tension and viscous dissipation [27], and Bellehumeur et al. incorporated temperature-dependent surface tension and viscosity into Pokluda et al.’s model. Although they did not model molecular diffusion, they found that the extruded material cooled too quickly for complete bonding. They also reported that the convective heat transfer coefficient greatly affects bond formation and neck growth, with less heat transfer leading to better neck formation. However, they modelled isothermal polymer sintering and did not consider heat transfer from the hot extruded material to the surrounding material. Bellini [28] performed extensive modelling of the entire FDM process with ceramic-filled filament using four different numerical simulations focusing on: the liquefier, the nozzle contraction, deposition on the printing bed and on stacked layers. This enabled the tracking of material temperature, swelling and filling as a function of the various printing parameters. It was found that the higher thermal conductivity of the filled material increases heat transfer from the liquefier to the printed material and improve the flow behavior.

### 2.3. Voids in FDM Printed Components

The strength of components produced by the FDM process differs from that of parts fabricated by conventional injection molding. The presence of voids and gaps between the individual layers reduces the layer-to-layer bond strength. The strength of fabricated components by FDM is compromised by significant voids and weak interlaminar bonding between layers. The percentage of void is depends on printing parameters and typically ranged from 4% to 18.5% [29,30,31]. Although the deposition filaments can be integrated into the adjacent deposition filaments by their gravity and the force of the printer’s stepper motor, the presence of significant voids between them greatly affects the mechanical properties of fabricated components. In addition, the extruded filament cools rapidly from the melting temperature to the chamber temperature, developing inner stresses responsible for a weak bond between two deposition filaments. This leads to a deformation between layer (inter-layer) and within layer (intra-layer) in the form of cracks, delamination or even part fabrication failure [32].

**Figure 5:**
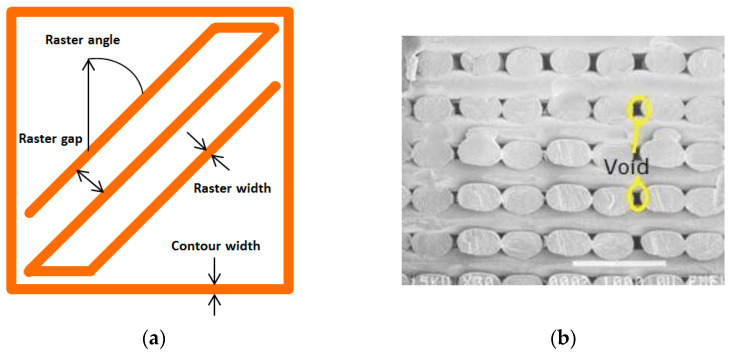
Raster gap voids [29]: (**a**) FDM process parameter [18] and (**b**) cross-sectional view of ABS printed part with 0/90 rasters. Reprinted with permission from [33], 2003, Emerald Publishing, Bingley, UK.

**Figure 6:**
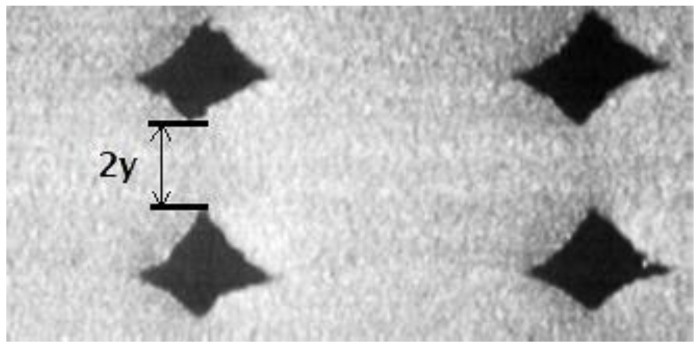
Partial neck growth voids, [29]: cross sectional view of an FDM laminate, where 2y is the neck growth between adjacent rasters. Reprinted with permission from [22], 2008, Emerald Publishing, Bingley, UK.

**Figure 7:**
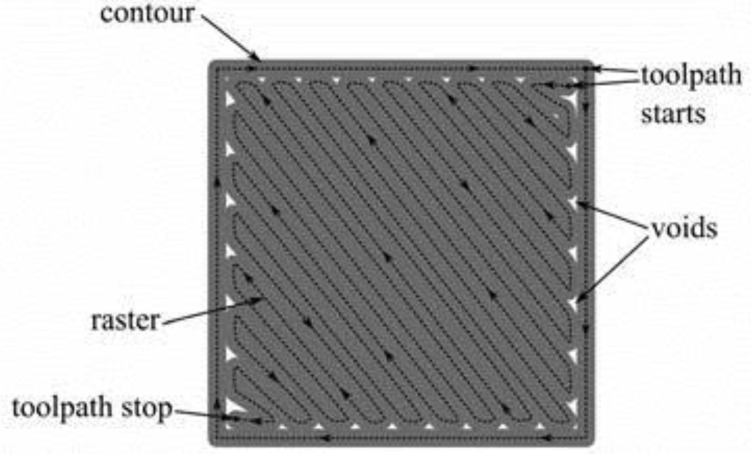
Sub-perimeter voids. Reprinted with permission from [34], 2015, Emerald Publishing, Bingley, UK.

**Figure 8:**
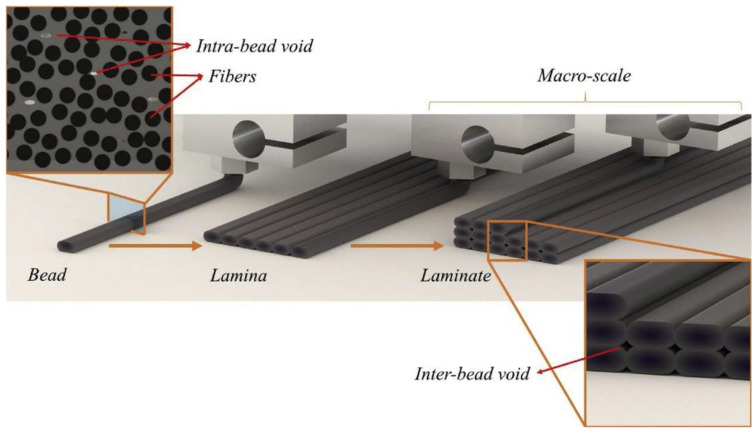
Intra-beads voids. Reprinted with permission from [35], 2020, Elsevier, Amsterdam, The Netherlands.

Voids in FDM printed part can be classified into five categories according to their formation mechanism: raster gap voids, partial neck- growth voids, sub-perimeter voids, intra-bead voids and infill voids [29]. Raster gap voids are formed by gaps between the raster surfaces and are visible, as shown in Figure 5. Partial neck growth voids are internal voids formed by incomplete neck growth between adjacent intra- and inter-layer rasters (Figure 6). This occurs when rasters solidify before coalescence is complete. Partial neck growth voids are a major contributor to voids in FDM prints. Due to physical limitations, sub-perimeter voids form in between turning rasters along the perimeter of the FDM layer. Even when the printer is set into 100% infill density, voids form between the blue wall lines and the infill zones, as shown in Figure 7. Intra-bead voids are specific to composites due to fiber loading, as shown in Figure 8. Finally, infill voids are voids in the infill depending on the infill pattern selected for printing the parts and can be controlled/adjusted.

### 2.4. Quantification of Voids

Density measurement, imaging technique, optical microscope (OM), scanning electron microscope (SEM) and CT scan are commonly used to study voids [29]. OM is widely used to study the meso-structures of printed parts, while SEM is often used to analyze microstructures. OM and SEM can both capture 2D images, with the right angle proportional to the FDM layers, to capture valuable data for the analysis, such as raster gap voids, partial neck growth voids, sub-perimeter and intra-bead voids. In contrast, a CT scan is a valuable tool for observing and investigating the effects of FDM voids in 3D. Moreover, CT scans can also be used to reconstruct 3D models of scanned specimens in great detail [29].

## 3. Printing Parameters

Four main factors affect the print quality of FDM printed parts: material, machine, printing process and environment [9]. Matrix and reinforcement materials are the main determining properties of the printed parts. Machine factors are mainly related to printer productivity. Nozzle temperature, heating mechanism, diameter and geometry influence the quality of the printer. The temperature is set according to the materials to be processed. The nozzle diameter affects the print resolution. Selecting the right temperature based on the material is tedious and significantly influences productivity and print quality. A high temperature results in better interlamellar bonding and less void space occupancy due to lower viscosity and better rheological properties, improving mechanical properties [36]. However, a too high temperature may affect printing quality, especially the dimensional accuracy. This section discusses the main parameters that are set before the printing process.

**Figure 9:**
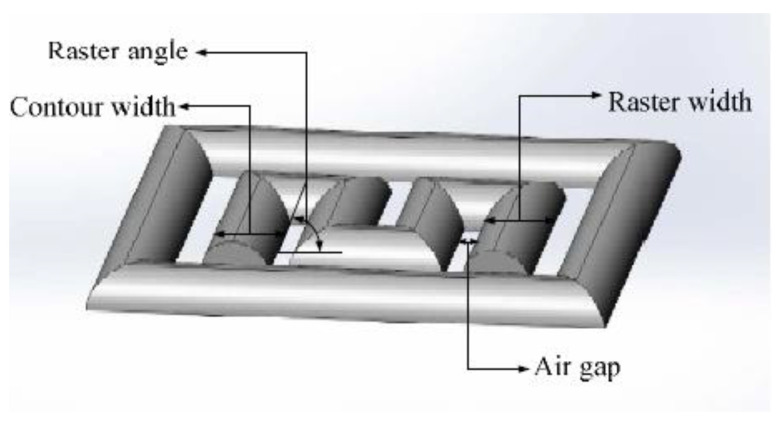
Raster angle, raster width and air gap. Reprinted with permission from [42], 2018, Emerald Publishing, Bingley, UK.

The FDM processed component primarily depends on three important control factors: namely the extruder, the processing and the structural, which are defined in Table 1 and illustrated in Figure 9. Selecting the optimal process parameters for printing will significantly improve mechanical performance, surface roughness and geometric accuracy. Based on previous research, it was found that the mechanical strengths such as tensile and flexural of parts printed by FDM are highly anisotropic [20,37], i.e., the performance of a material depends on the direction of printing. The printing speed has a minor effect on tensile strength but a significant effect on the production cost. A low feed rate increases interlamellar bonding but negatively affects productivity and increases production costs.

Air gap or raster-to-raster gap is the distance between two adjacent rasters on the same layer. The default value is usually zero, meaning the beads are touching. A positive gap means a gap between adjacent rasters or a negative gap, which means that the bead tracks overlap. With many slicing software that control the 3D printers, only the infill rate can be set, which represents the density of the printed pattern. Therefore, the actual positive or negative air gap must be determined or estimated, even if some specimens were printed with a setting of 100 infill. This affects the part as the air gap has been shown to be an important contributing factor in tensile strength [38].

Onwubolu et al. [39] investigated five significant process parameters: layer thickness, part orientation, raster angle, raster width and air gap. According to their results, the tensile strength was highest when the layer thickness is lowest, and the part orientation was printed parallel to the direction of the applied tensile force. Furthermore, a high raster angle with a low raster width and a negative air gap contributes to the increase in tensile strength, which was also observed by Dawoud et al. [40]. As shown in Figure 9, the raster angle, i.e., the angle of the deposited raster or bead relative to the horizontal direction, has a greater influence on tensile strength, toughness and ductility. Ning et al. [41] studied fiber orientations at 0°, 90° and ±45°. They found the latter had low tensile strength but better toughness and ductility due to poor interfacial adhesion between the matrix and reinforcing filament.

**Figure 10:**
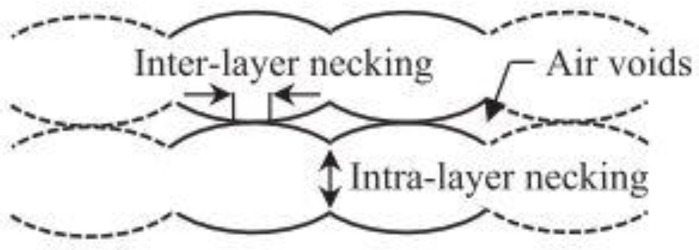
Formation of air void between printed layers. Reprinted with permission from [32], 2017, Elsevier, Amsterdam, The Netherlands.

Garg et al. [32] also studied the effects of layer thickness and raster angles on the failure of FDM parts under tensile loading. They found that to increase the mechanical strength, the printed part should be oriented so that the longest contours align the tension/stress force, which is confirmed by Onwubolu et al. [39]. As the layer thickness increases, the number of layers required to fabricate the specimen decreases, further decreasing the number of air voids in the interstitices. However, Onwubolu believes that a lower layer thickness increases the adhesion force in the layer-to-layer bond and hence improves the tensile strength. Figure 10 illustrates the presence of air voids in the deposited filaments. These air voids are the major cause of crack initiation and propagation in the specimen. Therefore, a lower number of air voids contributes to a higher strength of the printed parts.

Another investigation was carried out by Carneiro et al. [43] on the effects of build orientation, layer thickness, infill degree, and inclusion of fiberglass as reinforcement fiber. In this work, they also compared the results of FDM and specimens fabricated by compression molding. With an optimal selection of printing parameters and fiber reinforcement of the printed parts, the result shows that the strength is almost the same as the specimens fabricated by compression molding with less than 20%. They believed this result is not due to poor adhesion between the filaments but due to the existence of voids in the printed samples, which supports the results of Garg et al. [32].

On the other hand, infill pattern options such as honeycomb, hexagonal, triangular and solid structures significantly alter interfacial adhesion and interlamellar bonding. This parameter also affects the tensile, flexural, impact and compression properties. For instance, a solid infill pattern may have lower impact resistance than other pattern types because the free air gaps in these structures help absorb large impact shock [44]. Hui et al. [45] investigated how the different infill types, together with layering, affect the tensile strength and elastic modulus of carbon fiber (CF), glass fiber (GF) and Kevlar-reinforced Nylon composites. It was found that rectangular fill had the highest tensile strength, followed by hexagonal and triangular infill, respectively, but different behavior was noticed by another group of researcher [46].

Based on previous research and investigations, although optimization of processing parameters, such as build orientation, raster angle, infill degree, layer thickness, or feed rate to improve the mechanical performance of thermoplastics has been studied, the results are still poor due to the used of pure thermoplastics and the presence of air voids in each layer. Therefore, combining different materials to achieve the desired mechanical and functional properties is a promising way to solve these problems. Table 2 shows a compilation of previous studies on printing parameters’ effect on the mechanical properties of printed parts.

## 4. Fiber Reinforced Polymer Composite (FRPC)

The development of composites that are compatible with FDM printers has attracted a lot of attention. This is because composites promise better mechanical properties and performances compared to neat polymers. Many results in the development of new printable composites reinforced with particles, fibers or nanomaterials have already been demonstrated. Carbon black, platelets, chopped fibers and polymer fibrils are mixed with the polymer matrix and then extruded together during printing. However, the performance of these composites depends largely on the fiber orientation in the plastic and the fiber-volume-fraction (FVF). Parts manufactured with FDM from neat polymer have shown insufficient strength in load tests. This limits the range of applications in which FDM technology can be used for functional parts and not for prototypes.

Researchers have used fiber reinforced polymer composites (FRPCs) to overcome the aforementioned limitations in their work. In FRPCs, the material properties of a component are enhanced by combining reinforcing fibers and polymer matrix. Various fibers have been used for reinforcement, including chopped carbon fibers, carbon nanotubes, glass fibers, natural fibers etc. [72,73,74,75]. There are certain requirements that FRPC materials must meet in order to be processed by AM, namely:

1. Types of reinforcement and matrices;
2. Good fiber-to-matrix bonding;
3. Fiber homogeneity;
4. Fiber alignment;
5. Good interlayer bonding;
6. Minimal porosity.

The fiber reinforcement must be matched in size, shape and length to the part’s intended use. Both the matrix material, which holds the fibers in place, and the reinforcement must be compatible with the selected AM technique. A good bond between fibers and matrix is required at the fiber-matrix interface to transfer loads efficiently from the matrix, resulting in composites that follow the “rule of mixtures”. Fiber loading is also crucial to obtain AM composites with good mechanical properties. Mechanical properties such as elastic modulus increase with fiber loading at a low loading ratio but degrade after reaching an optimum value [76]. This phenomenon generally occurs due to poor wettability of the fiber with the thermoplastic, which results in a poor fiber-matrix interface.

Higher loading leads to an increase in viscosity and a decrease in flowability, leading to processability problems such as clogging of the nozzle. Furthermore, fiber reinforcement may cause negative effects on interlaminar bonding and the properties of printed parts. Based on previous research, interlamellar matrix regions between the reinforced fiber layers are critical regions that are highly prone to delamination when subjected to mechanical stress. Delamination can result from weak fiber-matrix bonding, which often leads to internal damage in composites, potentially leading to global failure of the component with reduced strength and stiffness [77]. Furthermore, porosity and weak interface bonding between fibers and matrix have been cited as a major problem for 3D printed fiber reinforced polymer composites [78]. Understanding the mechanism of filament bonding is important to further investigate how FRPC works to reduce voids and increase the strength of the interlaminar bond between the deposited filaments.

### 4.1. Synthetic Fibers vs. Natural Fibers

Various fibers were used as reinforcement for polymer composites and can be grouped under two categories: synthetic fibers and natural fibers [13]. Natural fibers were first used as reinforcement for polymers since 1936 [79] and were slowly replaced by synthetic fibers because synthetic fibers are usually much stronger than natural fibers. However, natural fibers re-emerged as reinforcing materials for polymer composites when environmental issues became more important in engineering applications. In FDM 3D printed polymer composites, both synthetic and natural fibers were used to reinforce polymers, although synthetic fibers are a more popular choice.

Synthetic fibers are commonly used as reinforcement for FDM printed composites, and the popular fibers are carbon fiber [41,80,81,82,83,84,85,86,87,88,89], glass fiber [81,90,91] and Kevlar fibers [81]. Other possible synthetic fibers are Graphene, CNTs [92], powder [93], copper powder [93] etc. Generally, synthetic fibers are added to polymer matrix during FDM 3D printing to enhance the mechanical properties of polymer composites, and plenty of works were reported previously [69,81,82,86,88,94]. In addition, synthetic fibers were also used to improve or alter thermal properties/thermal conductivities of FDM 3D printed polymer composites [95,96] and electrical properties [97]. A systematic review on synthetic fibers as reinforcement for polymer matrix was presented recently [98], although their review does not focus on FDM 3D printed polymer composites specifically.

Natural fibers are used as reinforcement to reduce the inorganic content in thermoplastic composites without compromising mechanical strength, ultimately improving biodegradability and reducing costs [18]. Common natural fibers used in FDM 3D printed polymer composites are jute [87], wood [99], harakeke/flax [100,101], bamboo [101], sugarcane and many more. Recent and systematic reviews on natural fiber reinforced polymer composites as feeders in FDM-Based 3D Printing were reported by researchers [18,102,103]. Natural fibers are a cheaper and greener alternative to reinforce polymer matrix during the FDM 3D printing process, but challenges such as fiber agglomeration, clogging in the nozzle, poor fiber-matrix interface, non -homogenous mixing etc., have to be investigated further. Furthermore, various treatments such as chemical and thermal are required to be applied to natural fibers to enhance the performance of natural fibers reinforced polymers. In addition, a different combination of polymer matrix and natural fibers requires different treatments and processes. As such, more research works are required to improve the performance of natural fibers reinforced polymers. Environmentally friendly engineering materials are getting more attention recently. Therefore, polymer composites produced by bio-based polymers such as PLA [99], soy-based resin [104,105] etc., and reinforced with natural fibers have great potential because they are biodegradable and environmentally friendly.

The advantages and limitations of synthetic and natural fibers as reinforcing materials for FDM printed polymer are summarized in Table 3.

### 4.2. Continuous vs. Discontinuous Fiber

Fiber reinforced polymer composite is a subcategory of fiber reinforced composites. Generally, fiber reinforcement can be categorized into discontinuous and continuous fibers according to critical fiber length [107]. Critical length *l*_c_ is the fiber length that allows applied load transfer to the reinforced fibers by the matrix, and depends on fiber’s ultimate strength *σ*_f_, fiber diameter *d*, and fiber-matrix bond strength or shear yield strength of the matrix *τ*_c_. Continuous fibers are referred to fiber with length more than 15 *l*_c_, and discontinuous fibers are fibers with length less than 15 *l*_c_ [107]. Nevertheless, some other researchers have slightly different definition. Krajangsawasdi et al. further classified short and discontinuous fiber, where short fibers are fibers shorter than critical length *l*_c_, and discontinuous fiber are those with length above critical length *l*_c_ [16]. Pruß and Vietor defined discontinuous fibers as fibers with fiber length less than 1 mm (0.04 in.), while continuous fibers are fibers with a length above 50 mm (2 in.) [108].

Besides the obvious motivation of improving mechanical properties, reinforcement can also be used to provide the material with additional functions such as electro-conductivity, thermal conductivity or biocompatibility. Kalsoom et al. [109] and Wang et al. [110] have provided a general overview of 3D printable composites; this paper instead focuses in more detail on the engineering aspects of FDM as a composite manufacturing method.

Conventionally, fiber-reinforced composites can be classified into: (a) continuous and aligned fiber composites, (b) discontinuous and aligned-fiber composites, and (c) discontinuous and randomly oriented-fiber composites, depending on the length and alignment of the fibers [107]. The major advantages and disadvantages are listed in Table 4.

#### 4.2.1. Continuous and Aligned Fiber Composites

The continuous and aligned fibers can reinforce composites in the intended direction but have no significant effect in the transverse direction. Conventional methods for producing continuous and aligned fiber composites are pultrusion, prepreg, and filament winding [107]. In terms of additive manufacturing, the FDM 3D printed ‘continuous and aligned fiber composites’ are being investigated by various researchers [80,81,82,86,88,94,97,112]. Previously, 3D printed continuous and aligned fiber composites were mostly printed using in-house developed or modified 3D printers [80,86]. The first commercial 3D printer capable of printing continuous and aligned fiber composites was developed by MarkForged. With the availability of commercial machines such as Markforged’s Markone, Marktwo 3D printers, research on FDM printing of continuous fiber reinforced thermoplastics (CFRT) composites is booming. Most of the recent research on FDM 3D printed continuous and aligned fiber composites uses Markforged’s Markone, Marktwo 3D printers [81,82,88,94,97,112]. Various types of continuous fibers, such as carbon fibers [80,81,82,86,88], glass fibers [81,94], and Kevlar fibers [81], have been used as reinforcement. In general, the FDM printed continuous and aligned fibers can have better electrical properties [97] and mechanical properties, such as tensile strength [81,82,86,88,94], flexural strength [86] if the printing parameters are properly selected. A systematic review of 3D printed continuous fiber polymer composites is presented by [113]. However, 3D printed continuous and aligned fiber composites are limited in terms of design freedom, as fiber placement is challenging and more voids are created, especially when printing complex shapes [19,88]. Design freedom is one of the main advantages of additive manufacturing over conventional manufacturing, and incorporating continuous fibers into FDM 3D printing, negates this advantage.

#### 4.2.2. Discontinuous and Randomly Oriented-Fiber Composites

Discontinuous fiber composites have a long history, and the first scientific publication dates back to 1936 [79]. Due to the nature of reinforced fibers and conventional fabrication methods, such as hand lay-up, resin transfer molding, etc., early fiber reinforced composites are mainly discontinuous and randomly oriented. FDM 3D printed discontinuous fiber composites are manufactured using composite filaments by commercial FDM 3D printers. Generally, discontinuous fibers were premixed with the polymer matrix as composite filament, and the composite filaments were then used in FDM 3D printing to produce discontinuous fiber composites. To date, more than 10,000 published papers have been found in Scopus using the keywords “additive manufacture” and “short fiber reinforced polymers”, and it is not possible to discuss them all here. However, most of these research papers focused on the mechanical or thermal properties of the composites. They did not report on the orientation of the fibers in FDM 3D printed discontinuous fiber composites. Nevertheless, research with FDM 3D printed discontinuous and randomly oriented-fiber composites have been reported by several researchers [91], although not all of them emphasized the orientation of the fibers.

One of the recent works with FDM 3D printed discontinuous and randomly oriented-fiber composites was reported by Zhao et al. [91]. They compared the tensile properties of 3D printed CNT-short glass fiber (SGF) reinforced PLA composite with the tensile properties of 3D printed PLA, SGF/PLA, and found that both composites are better than neat PLA in terms of tensile strength and tensile modulus. In addition, CNT-SGF /PLA composite has a higher tensile strength than SGF/PLA composite. From the SEM images of the fracture surfaces of the composite specimens, they found that the fibers in the composites are randomly oriented. Su et al. reinforced polyamide with reclaimed carbon fiber in four different weight percentages (10%, 20%, 30%, 40%). They found that the fibers were better aligned at low fiber contents (10–20%) and had no significant alignment at 40%. They concluded that the tensile performance of the reclaimed carbon fiber reinforced polyamide composites (rCF/PA) largely depended on the fiber content and orientation, with higher fiber content and aligned fiber being able to improve tensile strength. All composites, including rCF/PA with 40 wt% and non-aligned fiber performed better than neat PLA [114].

#### 4.2.3. Discontinuous and Aligned-Fiber Composites

**Figure 11:**
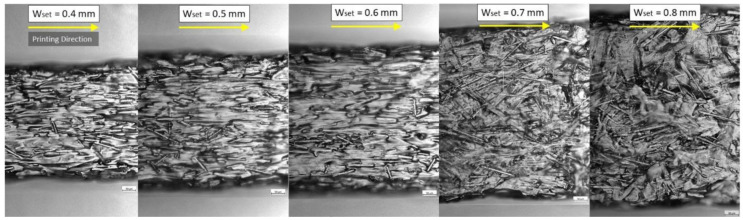
Fiber orientation at different extrusion width (W_set_), where fibers were more aligned in a narrow extruder compared with a wider extruder. Reprinted with permission from [118], 2022, Elsevier, Amsterdam, The Netherlands.

Discontinuous and aligned fibers are an alternative to continuous fibers in 3D printing of polymer composites, with the advantage of better design freedom. Early research on discontinuous and aligned-fiber composites (also named as aligned discontinuous fiber thermoplastic) produced by non-additive manufacturing processes was summarized by Such et al. [115]. Although the manufacturing methods for 3D printed FDM 3D printed discontinuous and aligned-fiber composites are different from the conventional make discontinuous and aligned-fiber composites, the motivations for reinforcing polymers with discontinuous and aligned-fiber are similar. In general discontinuous and aligned-fiber are added to polymers for three main reasons: (1) to improve mechanical, thermal, or electrical properties in the desired direction, (2) to reduce the cost and complexity of manufacturing compared to composites with continuous fibers, and (3) enabling design freedom or complex geometries [20,79,115,116]. FDM 3D printed discontinuous and aligned-fiber composites are mainly manufactured using composite filaments by commercial FDM 3D printers. The discontinuous fibers were aligned by shear (referred to as shear-induced alignment or flow-induced alignment), where the shear force between a nozzle and the molten material forces the fibers to align in the direction of extrusion or flow [20,117]. Furthermore, the orientation of fibers is affected by experimental extrusion width, where experimental extrusion width depends on extrusion temperature, speed and width. Fibers were more aligned in a narrow extruder than in a wider extruder [118], as shown in Figure 11. One of the first published papers on FDM 3D printed discontinuous, and aligned-fiber composites was by Tekinalp et al. [76]. They fabricated the carbon fiber reinforced ABS filament and used the filament with a commercial FDM 3D printer. They applied the method of Bay and Tucker [119] to characterize the fiber orientation in the printed part and found that the carbon fibers in the printed parts are mainly oriented in the load-bearing direction. They concluded that the carbon fibers could increase the strength and modulus of both the FDM printed and compression molded samples, but the FDM samples have significant voids [76].

Jia et al. fabricated graphite flakes reinforced PA6/POE-g-MAH/PS composite with an FDM 3D printer and verified by microscopy that the graphite flakes were aligned along the through-plane direction (parallel to the x-y plane) via microscopy. With this designed composite, they were able to improve the thermal conductivity of the polymer [120]. However, they also pointed out that the presence of voids in FDM- printed composites affects the through-plane thermal conductivity of the composites. Papon and Haque investigated fracture toughness of 3D printed carbon fiber reinforced PLA composites with different fiber content (3 wt.%, 5 wt.%, 7 wt.% and 10 wt.%), manufactured by two different nozzle shapes (circular and square) [121]. The square shape nozzle was custom-made to improve the contact area and inter-bead void. The fibers are mostly aligned in the extrusion direction, but they did not report how nozzle shape affects the fiber orientation. Their experimental results show that the fracture toughness increased with fiber content from 0% to 5%, at both layer orientation of 45°/−45° and 0°/90°. The print layer orientation of 45°/−45° and 0°/90° has no major different in fracture properties. Furthermore, parts printed by a square nozzle have better fracture toughness than parts printed by a circular nozzle because less void is produced in parts produced by the square nozzle.

Researchers at the University of Bristol developed a method named High Performance Discontinues Fiber (HiPerDIF) to manufacture discontinuous and aligned-fiber composites [122] and investigated the performance of composites produced with this method [116]. Generally, the fibers were suspended in a liquid medium (water), and the orientation of fiber was controlled by the orientation head [122]. With this method, they fabricated discontinuous and aligned-fiber epoxy composites using carbon fiber [122] and recycled carbon fibers [123]. They reported that the mechanical properties of composites are proportional to the fiber lengths [123]. To expand the HiPerDIF technology to additive manufacturing/FDM, Blok et al. have identified 4 different polymers (ABS, PLA, Nylon, PETG) as the potential polymer matrix materials to be reinforced with high performance discontinues and formed the feedstock materials for FDM. The four polymers were selected based on 14 factors. They fabricated the composite tapes using an in-house consolidation method, where the HiPerDiF fiber was sandwiched between two layers of polymer matrix films of 0.125 mm.

They proofed that aligned discontinuous fiber composites produced using HiPerDIF technology are better than currently available short fiber thermoplastic. Furthermore, the composite fabricated with HiPErDIF technology has comparable mechanical behavior compared with continuous fiber composite but with better manufacturing flexibility [89]. Krajangsawasdi et al. recently extended their work by fabricating 3D printer filament using ADFRC fiber to reinforce PLA thermoplastic. They managed to produce HiPerDiF-PLA filament and also identified the optimal printing parameters of their newly developed filament. They compared the mechanical properties of the HiPerDiF-PLA printed parts with PLA, PLA-short carbon fiber, PLA-continuous carbon fiber, and Markforged continuous carbon fiber [116], and they concluded that HiPerDiF-PLA outperformed other PLA composites in terms of mechanical performance.

## 5. Manufacturing Techniques of Fiber Reinforced Polymer Composites

**Figure 12:**
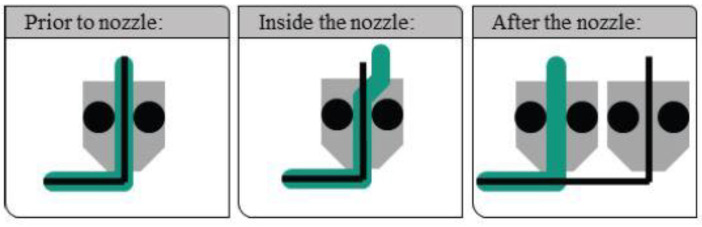
Methods of continuous fiber implementation based, grey areas are the nozzles, green strings are the extruded thermoplastics, and the black strings are fibers. Reprinted with permission from [124], 2017, Elsevier, Amsterdam, The Netherlands.

When manufacturing fiber-reinforced polymer composites, the techniques used to embed the fiber into a thermoplastic matrix influence the mechanical properties of the printed parts. There are at least three different ways of embedding fibers, taking into account the timing and location of the embedding of fibers [108]. Figure 12 illustrates the three techniques for embedding fibers in the matrix used by researchers to reinforce continuous fibers.

(i) Method 1 (M1): embedding before the printing process.

Prefabricated composite, which is the filament itself, is a composite.

(ii) Method 2 (M2): embedding in the nozzle.

The fiber embedding can take place in the extruder itself.

(iii) Method 3 (M3): embedding on the component.

This method requires two or more independent extruders with an independent nozzle.

### 5.1. Method 1 (M1): Embedding before the Printing Process

**Figure 13:**
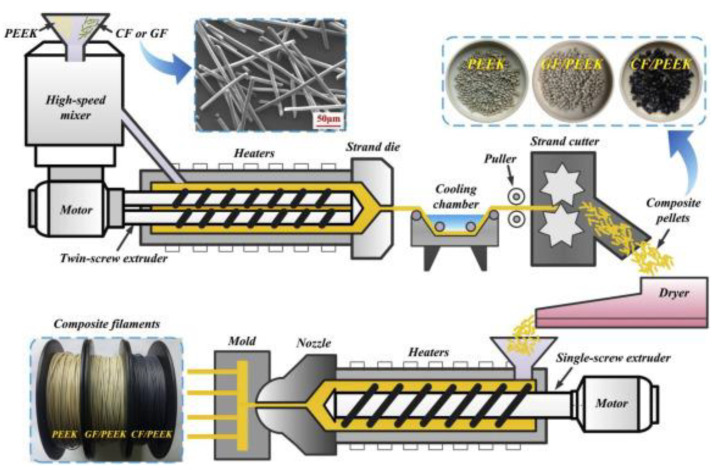
Preparation process of composite filaments, for M1 embedding before the printing process Reprinted with permission from [125], 2020, Elsevier, Amsterdam, The Netherlands.

M1 is the method most used by researchers because it is the most straight forward approach and does not require major modifications to the machine. The polymer matrix and reinforcement are premixed before 3D printing in the form of 3D printer composite filaments. Commercial composite filaments such as carbon fiber/ PETG, carbon fiber/PLA, carbon fiber/Nylon etc., are available in the market. However, only a limited type of fiber (mostly carbon fiber) was used to reinforce commercial composite filaments, and other fibers’ potential is under investigation. Various researchers studied combinations of different matrix and different reinforcements. Usually, the composite filaments were fabricated in-house, following the processes described by [41,125] and shown in Figure 13. Although the exact procedures used by different researchers might not be identical, the treated fibers and polymer matrix are generally mixed in a mixer or blender and then fed into an extruder (twin-screw extruder or extruder). The composites normally go through the second extrusion process to obtain better matrix distribution and reinforcement by supplying fiber matrix. The prefabricated composite filament has a constant fiber volume ratio.

Discontinuous fibers, or short fibers in different sizes, were premixed with a polymer matrix to form composite filaments for FDM 3D printing. Some of the materials studied are nanoscale single-walled carbon nanotube (SWCNT) [126], vapor-grown carbon fiber (VGCF) [127], graphene [92], micrometer-sized metal powders of copper and iron, millimeter long chopped fibers of thermotropic liquid crystalline polymers (TLPs) [128], glass [129] and carbon [76]. Natural fibers such as harakeke and hemp have also been used as reinforcement [130].

For nanofiber-reinforced polymers made with FDM, Shofner et al. [127] combined vapor-grown carbon fibers (VGCFs) with ABS copolymer to create a composite filament for use with FDM. The dispersion, porosity and fiber alignment issues were also investigated to prevent agglomeration when mixing VGCFs with ABS. The results show that the tensile strength and modulus of VGCF-filled ABS are, on average, 39% and 60% higher, respectively, than those of the neat ABS. On the other hand, the storage modulus measurements from the dynamic mechanical analysis indicated that the stiffness increased by 68%.

**Figure 14:**
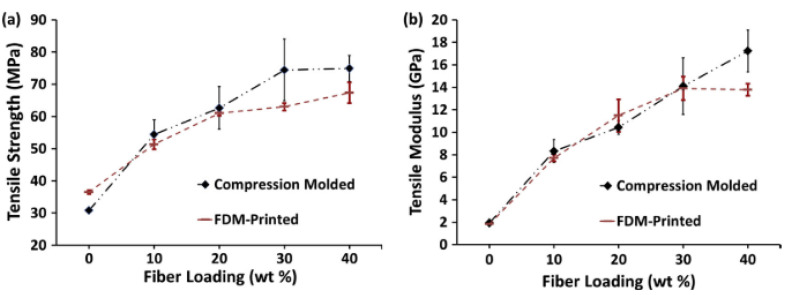
Typical tensile stress-strain curves for specimens with different carbon fiber contents (**a**) tensile strength, and (**b**) modulus, of ABS/CF composites. Reprinted with permission from [76], 2014, Elsevier, Amsterdam, The Netherlands.

Zhong et al. [129] conducted experiments to investigate the processability of short glass fiber reinforced ABS matrix composites with three different glass fiber contents used as feedstock filaments in FDM. The results showed that glass fiber could significantly improve the ABS filament’s tensile strength and surface rigidity. The effect of fiber content on the mechanical properties of printed parts is another interesting research topic. Similar but not identical work was also reported by Wang et al., where carbon fiber and glass fiber with different fiber contents were mixed with PEEK to form composite filaments [125]. Both CF/PEEK and GF/PEEK composites were reported to have better tensile and flexural strengths than the neat PEEK, and GF/PEEK was stronger than CF/PEEK in terms of tensile and flexural strengths. They also highlighted that composite with higher fiber contents had more porosity (voids) and lower mechanical properties (tensile strength, flexural strength, impact strength and ductility). Tekinalp et al. [76] demonstrated that the short carbon fibers (0.2–0.4 mm) reinforced ABS composites produced by FDM showed an increase in tensile strength and modulus with increasing fiber content, with a maximum increase of 115% and 700%, respectively, at a fiber content of 40 wt%. However, the tensile strength of the FDM printed specimen is still lower than the tensile strength of compression molded specimen, especially at higher fiber loading. Tensile strength and modulus measurement of specimens prepared by both FDM and compression molding are shown in Figure 14.

**Figure 15:**
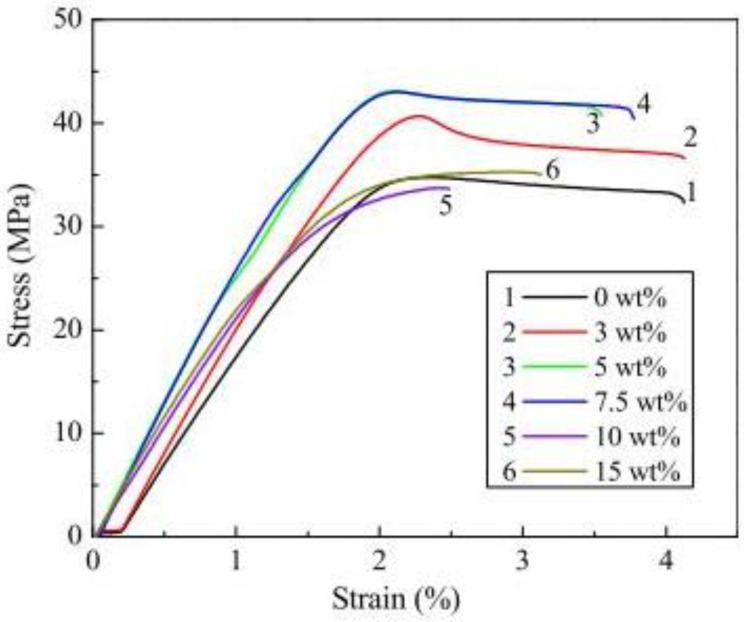
Typical tensile stress-strain curves for specimens with different carbon fiber contents. Reprinted with permission from [41], 2015, Elsevier, Amsterdam, The Netherlands.

Ning et al. [41] studied the use of thermoplastic ABS composites with different percentages of added carbon fibers (CF) of various sizes in an FDM printer. Their observation showed that the addition of CF increased the tensile strength and Young’s modulus of the plastic compared to pure plastic specimens. Moreover, the CFRP specimens enriched with longer carbon fibers (150 µm) had higher tensile strength and Young’s modulus as well as lower toughness and ductility than the specimens with shorter carbon fibers (100 µm). They also studied the effect of fiber content on the mechanical properties of the FDM printed ABS/carbon fiber composites. The best performance of the printed parts was obtained at a fiber content of 5 wt%. Higher fiber content deteriorated the performance of printed parts due to the higher porosity. Figure 15 shows that the highest mean value was found at 5 wt% CF with 42 MPa, and the lowest at 10 wt% with 24 MPa, which was almost the same with neat ABS plastic. Ning et al. [73] also reported the fabrication of M1 short CFRP specimens using an FDM machine (Creatr, Leapfrog Co., Alphen aan den Rijn, The Netherlands) and a composite filament (FilaBot Co., Montpelier, WT, USA) with a diameter of 1.75 mm that contained 5 wt% chopped CF in ABS thermoplastic matrix. Their work evaluated the mechanical properties of parts printed at four different process parameters: Nozzle temperature, infill speed, raster angle and layer thickness. The objective was to find the best parameters to improve the tensile strength of the parts.

Liao et al. [131] investigated the mechanical performance of carbon fiber (CF) with 15–20 mm fiber length reinforced polyamide 12 (PA12) samples with various carbon fiber loading, 2 wt%, 4 wt%, 6 wt%, 8 wt% and 10 wt%. The results show that the tensile strength and flexural strength of 10 wt% CF/PA12 composites are enhanced by 102.2% (tensile strength from 46.4 MPa to 93.8 MPa) and 251.1% (flexural strength from 35.6 MPa to 124.9 MPa), respectively. To achieve maximum improvement of mechanical properties, the proportion of fibers varies considerably in the different cases of the study, mostly because the conditions for fiber distribution and interfacial bond strength differ greatly from case to case. So far, the added content of fibers is up to 40 wt%, and the composites with more fibers cannot be printed due to nozzle clogging problem. In addition, composites with higher fiber loading are difficult to process into continuous filaments for FDM due to loss of toughness. Therefore, the properties of the resulting composites are limited by the low fiber content. Applying plasticizers and compatibilizers could be a possible way to improve the feedstock processability [129].

Most of the reported research utilized short fiber in producing polymer composites by using method 1 (Embedding before the printing process) and reported research on FDM 3D printed composites with continuous fiber is relatively scarce. FDM 3D printed composites with continuous fiber are mostly produced with self-manufactured continuous carbon fiber reinforced filament [132,133,134]. Hu et al. designed a device to manufacture continuous fiber reinforced thermoplastic (CFRTP) filaments, where molten resin was squeezed into continuous carbon fiber to form continuous carbon fiber prepreg filament [132]. They optimized the printing parameters (printing temperature, printing speed, layer thickness) during printing continuous fiber reinforced PLA and found the optimized composite had better flexural strength than the neat PLA. Similar manufacturing technique was utilized by Zhang et al. [134] and Uşun et al. [133] to produce continuous fiber reinforced filament. Zhang et al. produced two different composites, the continuous carbon fiber reinforced PLA (CCF-PLA) and the continuous carbon fiber reinforced Nylon (CCF-Nylon). They found that CCF-PLA had higher tensile and bending strength than neat PLA and short carbon fiber reinforced PLA. Similarly, CCF-Nylon had higher bending and tensile strength than neat Nylon. Uşun et al. fabricated continuous fiber-reinforced thermoplastic (CFRTP) filaments with a melt impregnation line and later printed 3D parts with the CFRTP filaments. Their experiments showed CFRTP CF-PLA composites with 40% CF have higher tensile and flexural strength than composites with 22% CF and 33% CF [133]. Recently, a refined manufacturing technique was proposed to manufacture continuous fiber reinforced thermoplastic filament [135] and the manufactured filament has better prepreg quality and volume fraction. However, they did not report the mechanical properties of 3D printed part by using the new filament.

### 5.2. Method 2 (M2): Embedding in the Nozzle

**Figure 16:**
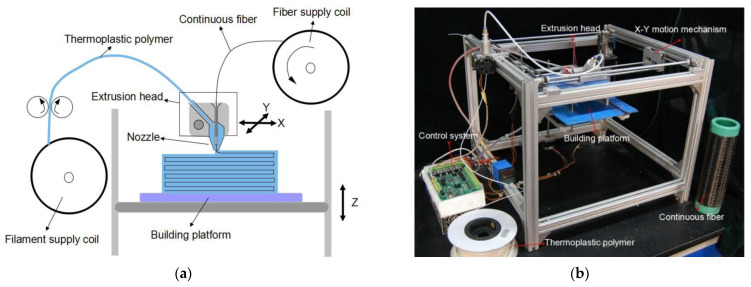
Printing composites by method 2 (M2): embedding in the nozzle. FMD 3D printing process using continuous filament with a printing head of a single nozzle (**a**) schematic diagram (**b**) actual experimental setup Reprinted with permission from [86], 2017, Emerald Publishing, Bingley, UK.

In method 2, both polymer matrix and reinforcing fiber are mixed in the printing nozzle during the printing process. Embedding the fiber to the polymer matrix in the nozzle during the printing process requires two material supplies, one for the polymer matrix and another for the reinforcement (fiber). The extrusion nozzle receives both the thermoplastic polymer and the continuous fiber, and the continuous fiber is fed through the core of the nozzle, as shown in Figure 16. When the nozzle is heated, infusion of the matrix occurs, and the molten thermoplastic material is deposited along with the reinforcing filament. Research machines mostly produced polymer composites fabricated with this method, as no commercial 3D printer that provides a similar function is available in the market. Fidan et al. [15] mention in their review that in the fabricating of composites, the selection of matrix and reinforcement must be compatible physically (good adhesion), chemically (matrix and fiber must not react chemically) and thermally (similar thermal behavior; coefficient of thermal expansion).

In the case of continuous fibers, the reinforcing fibers are then supplied as a dry roving. The thermoplastic matrix is added separately. This allows adjusting the fiber volume ratio by machine control during the printing process. This enables the same nozzle to deposit neat plastic. However, these advantages can be offset by additional challenges. For example, the infiltration process of dry roving without air inclusions must be carefully controlled, while the extrusion and deposition processes must run simultaneously. Furthermore, handling a dry roving with its non-rigid properties is much more difficult than handling a pre-impregnated one. Fabrication of specimen by using method M2 also was reported by Yang et al. [86] and Tian et al. [80]. They presented a 3D printing equipment with a novel composite extrusion head that can continuously process CF with ABS and PLA, respectively. Yang et al. developed a novel composite extrusion head in which dry carbon fiber is fed through a melt pool of ABS. This increased the in-plane mechanical properties by a factor of 205, but a limiting factor was the interlaminar shear properties of the printed part. Research about the implementation of continuous fibers inside the nozzle with different machine setups was reported by Prüß and Vietor [108], where they designed and fabricated an adapted FDM print head with 1 port for fiber and 2 ports for polymers.

A novel technique called continuous lattice fabrication (CLF) was proposed by Eichenhofer et al. [136]. The CLF head consists of a two-stage, pultrusion-extrusion system. They reported an increase in tensile properties of carbon fiber reinforced PA12 composites, which can achieve a tensile strength of 560 MPa and elastic moduli of 83 GPa along the fiber direction. The material properties obtained are comparable to those of high-performance aluminum alloys, which have a tensile strength of 540 MPa and tensile modulus of 72 GPa [137]. The technique involves a softening cycle procedure [137] that attempts to maximize the mechanical properties of the printed composites by minimizing the residual void content. Matsuzaki et al. [87] printed continuous fibers (straight carbon fibers or twisted jute fiber yarns) by feeding them through a nozzle simultaneously with a thermoplastic filament (PLA) serving as a matrix. They reported a strength and stiffness of 195 MPa and 10.5 GPa, respectively, due to a low V_f_ of 6.6%. This technique also showed uneven fiber distribution as the fiber was not pre-impregnated into the matrix.

### 5.3. Method 3 (M3): Embedding on the Component

**Figure 17:**
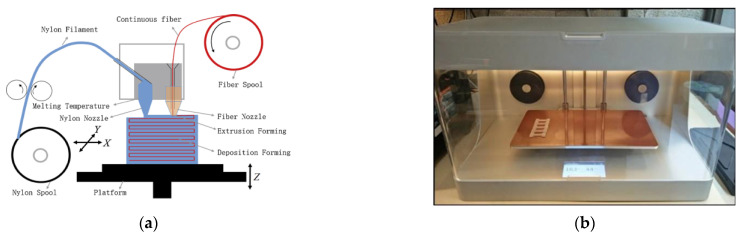
Printing composites by method 3 (M3): embedding on the component. (**a**) Schematic diagram Reprinted with permission from [138], 2019, Elsevier, Amsterdam, The Netherlands, (**b**) actual experimental setup-A Mark One Composite 3D printer Reprinted with permission from [81], 2017, Elsevier, Amsterdam, The Netherlands.

Composites produced by method 3 require two or more independent extruders, each with an independent nozzle, to deposit both polymer matrix and reinforce fiber on the printer bed, as shown in Figure 17. Several research that implemented the ‘fibers after the nozzle method’ used 3D printers manufactured by Markforged [46,81,97,138,139]. Markforced, with its MarkOne, MarkTwo and MarkX printers, is the first (and only, to the best of our knowledge) company that produced commercially available printers which can manufacture continuous FRPC [140]. Research with M3 process was presented by Dickson et al. [81], who report the fabrication of continuous carbon, fiberglass and Kevlar fiber reinforced polymer composites using a Markforged Markone 3D printer. Naranjo-Lozada et al. [46] compared the tensile performance of Nylon composites fabricated by two different fiber insertion methods; M1 with chopped carbon fiber and M3 with continuous carbon fiber by a Markforged Marktwo printer. Three test setups were carried out to compare the tensile strength of pure nylon, chopped carbon fibers and continuous carbon fibers. In addition, printing parameters such as infill density, infill pattern, fiber volume fraction and build orientation were also investigated. Naranjo-Lozada et al. concluded that increasing the fiber concentration and length at optimal printing parameters improves the tensile strength of printed parts. The results suggest that the initial point of application of the reinforcement fiber affects the tensile properties of the specimen. Mei et al. printed carbon reinforced Nylon composites with Marktwo printer at different fiber angles, and they concluded that the sample printed with mixed isotropic fiber angle [0°/45°/90°]^2^ is stronger than samples printed at fiber angles [30°/45°/60°]^2^ and [15°/45°/75°]^2^. In addition, they also reported that hot-pressed composites have higher strength compared to their non hot pressed counterpart [138].

Non-commercial printers or self-developed methods also produced composites produced by method 3. Implementing the fibers after the nozzle directly into the print job is done by Mori et al. with their ‘dieless forming’ method [141,142], where carbon fibers were placed manually on FDM printed acrylonitrile butadiene styrene (ABS) plate. Mori et al. have conducted experiments with manually deposited continuous fibers between plastic layers made with FDM [141]. Their research shows that the carbon fibers were entirely pulled out from a ruptured tensile test specimen, and the fibers had little effect on the resulting tensile strength due to weak fiber adhesion. They recommend a thermal post-process to bond the fibers to the matrix. This enables the specimens to reach almost twice the strength. With an additional thermal bonding, the test specimen reached about twice the tensile strength of unreinforced specimens [142]. Since the tensile strength without fibers was only 11 MPa, about a quarter of common ABS values, the FLM process quality, in this case, is more than questionable. 

Baumann et al. [124] used three different fiber implementation concepts (direct overprint, hypodermic needle, solvent) to fabricate polymer composites. They found out that direct overprint is the best method among the three. They also reported that the M3 technique significantly increases tensile strength and elastic modulus for different cases of continuous carbon fiber reinforced polymers. This study showed their processes’ potential for producing functional parts for engineering applications.

This classification is important because the properties of the part depend not only on the amount, often measured as the volume fraction of the reinforcing fiber, but also on the manner in which the fibers are integrated into the matrix material. Currently, FDM 3D printers that can fabricate composite with the M3 method are commercially available for continuous fibers, whereas only M1 is available for short fibers, as summarised in Table 5. To give an idea of the current performance in terms of fiber reinforced composites and the mechanical performances for parts produced with FRPC using the FDM process, this section summarizes a large collection of data on the mechanical properties and methods of fiber implementation for various fiber reinforced material systems. The mechanical characterization of printed fiber-reinforced polymers compared to neat polymer material is the primary focus of researchers publishing research in the field of FDM with composites. Table 6 summarizes previous research on different fiber implementations and the effect on the mechanical performances of FDM printed parts.

## 6. Opportunities for Future Developments

Currently, three methods (M1, M2, M3) are commonly used to fabricate fiber reinforced polymer composites. As discussed in the previous section and Table 5, most researchers have produced composites where the short fibers have been reinforced before the printing process (M1), and the short fibers added in this way are unable to reinforce intralayer and interlayer adhesion strength effectively. Embedding short fibers on the component (M3) may affect the intralayer and interlayer adhesion of deposited parts, but no previous work in embedding short fibers on the component is reported. Hence, embedding short fibers on the component is worth further investigation, and a new fabrication technique/machine is required to achieve this.

Furthermore, due to better environmental awareness, green composites such as fiber-reinforced bio-polymer composites produced by bio-based polymers and natural fibers, require further studies. Examples of popular bio-based polymers are PLA, biodegradable polybutylene adipate terephthalate (PBAT), polybutylene succinate (PBS), etc. [153,154]. Previously, most research focused on synthetic fiber reinforced PLA, and currently, some works on natural fiber reinforced PLA are reported [103,152]. The potential of FDM printed fiber reinforced composites formed by other bio-based polymers or bio-degradable biopolymers (as matrix) and natural fiber (as reinforcement) should be explored. Similarly, bioactive particles integration within the FDM can be explored [155,156].There is also a need to develop better numerical models with a focus on: (i) the manufacturing process of printed parts to reduce development costs, optimize the deposition parameters and provide a better scientific understanding of the fiber reinforced FDM process, based on current research in simulation of FDM manufacturing process [157,158]; and (ii) developing models to understand the deformation and failure mechanism of printed parts under mechanical and/or thermal loading conditions [32,151,159]. Some of these models can incorporate hardening, pore closure, creep and the plasticity of polymeric materials [160,161,162].

Next, most of the previous research focused on the fundamental aspect such as mechanical, thermal, or electrical properties of FDM 3D printed fiber reinforced polymer in the form of standard test specimens instead of the actual product, despite some works on actual products being reported [64,163,164]. Further research on the performance of actual fiber reinforced composite products fabricated via FDM 3D printing is necessary before FDM 3D printed fiber reinforced composite can be adopted widely in product design.

## 7. Conclusions

The Fused Deposition Modelling method can significantly affect manufacturing industries and additive manufacturing technology. The ability to produce functional parts directly from a commercial 3D printer with controllable properties has created a huge rush for new developments and research in this field. This paper provides an overview of FDM 3D printing and the formation of voids in 3D printed parts. FDM printing of fiber reinforced polymer composites can be an ideal method to improve the mechanical properties, thermal properties and electrical properties of FDM-printed parts. Both synthetic fibers and natural fibers of different lengths can be used as reinforcement for polymer matrix to produce different outcomes. On top of fiber type and length, fiber implementation technique is another factor that can affect the performance of composites. Currently, three techniques (M1, M2 and M3) are commonly used to fabricate fiber reinforced polymer composites. Embedding short fibers on the component is not explored and required further investigation. In addition, 3D printed green composites with bio-based polymer or biodegradable polymer and natural fibers are also worth for further investigation. Lastly, to optimize the application of FDM printed fiber reinforced composites, numerical models for both printing process and failure mechanism have to be developed, and testing on 3D printed actual products have to be conducted.

## Figures and Tables

**Table 1:** Definitions of fundamental printing parameters for FDM [18].

Parameters		Description
Extruder geometry	Nozzle diameter	Size of the exit orifice of the extruder
	Filament diameter	Size of the filament required by the extruder
Processing	Melt temperature	Temperature of the molten material exiting the extruder
	Bed temperature	Surface temperature of the workspace plate
	Printing speed	Speed of the material deposition
Structural	Layer thickness	Thickness of the layer deposited by the nozzle
	Infill pattern	Internal structure of the printed component
	Infill density	Material percentage filling the component apparent volume
	Raster angle	The angle between the deposed material and the x-axis
	Raster gap	The distance between two contiguous paths on the same layer
	Build orientation	Basic print build either upright, on-edge and flat

**Table 2:** Examples of previous research in printing parameters aspect.

Author	Material	Printing Parameters	Results	Ref.
Anitha et al. (2003)	-	Road width, layer thickness, deposition speed	Results showed that the best possible values of layer thickness, road width and the deposition speed were 0.3556 mm, 0.537 mm and 200 mm/s	[47]
Sood et al. (2009)	-	Layer thickness, build orientation, raster angle, raster width and air gap	Strength improves when, increase layer thickness, high raster angle and zero air gap	[48]
Nunez et al. (2015)	ABS-plus	Infill density, layer thickness	Results showed that low layer thickness and high infill densities were favourable for better surface finish. High layer thickness and infill densities tend to improve the dimensional accuracy	[49]
Kaveh et al. (2015)	ABS, PLA, HIPS	Extruder temperature, flow rate, feed rate, raster width, raster angle	Found that at constant feed rate 16 mm/s. 210 °C was the optimum temperature. Optimum raster width for each layer thickness cause to eliminate air gap between rasters	[50]
Baich et al. (2015)	ABSplus-P430	Infill pattern, infill density	As expected, lowest infill density enabled cost saving but mechanical properties were seen to deteriorate	[51]
Harpool et al. (2016)	PLA	Infill pattern (rectilinear, diamond, hexagonal, solid)	Results showed hexagonal pattern with infill density of 15% gave the highest strength, while solid pattern was the weakest even at 100% infill density	[52]
Behzadnasab et al. (2016)	PLA	Printing temperature	When increasing nozzle temperature from 180 °C to 240 °C the strain at break value increases from 34 MPa to 56 MPa which is close to the value of the injected moulding sample. However, a higher set nozzle temperature caused in polymer degradation	[53]
Alafaghani et al. (2017)	PLA	Infill pattern, printing speed, infill density, build direction, layer thickness, nozzle temperature	Found that, to improve the mechanical performance of printed parts; higher extrusion temperature and larger layer thickness are needed in addition to suitable building direction, that makes the layers and the load direction in parallel plane	[54]
Cristian et al. (2017)	ABS	Raster angle, infill density, infill pattern, build direction, nozzle temperature	Findings showed an increase of Young’s modulus with the percentage increase of infill density, 0^o^ and 90^o^ and raster angle provided the greatest strength	[38]
Rahman et al. (2018)	ABS	Bed temperature, nozzle temperature, print speed, infill, layer thickness, number of loopa	Finding showed the optimum parameter setting for bed temperature (110 °C), nozzle temperature (220 °C), print speed (55 mm/s), infill (15%), layer thickness (0.2 mm) and number of loops (1)	[55]
Korga et al. (2019)	ABS	Infill percentage	100% infill samples have the best impact strength No significant trend of impact strength was reported for samples with infill percentages from 10% to 90%	[56]
Zakaria el al. (2019)	PLA	Level of print head, printing orientation, layer thickness	Tensile and flexural strengths were optimized based on Taguchi’s method and Analysis of Variance Print head has major influence on the tensile strength and flexural strength	[57]
Bakradze et al. (2020)	PA, ABS	First-later bead height, first layer bead width, extrusion temperature, bead height, bead width, extrusion multiplier, printing speed, extrusion temperature, retraction distance, retraction speed, bridging extrusion multiplier, bridging printing speed	A heuristic model was created to optimize printing time, material consumption, and tensile behavior based on several printing parameters	[58]
Sammaiah et al. (2020)	ABS	Infill density, layer heights	Optimum surface roughness is obtained with higher infill density and lower layer heights	[59]
Sneha et al. (2020)	PLA-bronze (PLA-Bz)	Nozzle temperature, bed temperature, layer height	Flexural and compression strengths are influenced by nozzle temperature, and less influenced by bed temperature and layer height	[60]
Ramesh et al. (2021)	Nylon	Print speed, layer height, fill density	Tensile strength, impact strength, flexural strength, and hardness are maximum at 100% infill density Infill density has more influence on mechanical properties than print speed and layer height Print speed has least influence on mechanical properties	[61]
Giri et al. (2021)	PLA	Build orientation, layer thickness, cooling rates	Tensile strength is depending on interaction effect of build orientation, cooling rates and layer thickness	[62]
Hikmat et al. (2021)	PLA	Build orientation, raster orientation, nozzle diameter, extruder temperature, infill rate, number of shell, extruding speed	Tensile strength is mainly affected by three parameters (build orientation, nozzle diameter, and infill density Optimum parameters were determined	[63]
Muflikhun et al. (2021)	PLA	Build orientation, infill density	Carabiner was printed in three printing orientation and five different infill density 100% infill and X orientation produced the best strength	[64]
Patil et al. (2021)	PLA	Infill pattern, infill percentage, printing speed, layer thickness	Surface roughness, printing time and length of filament consumed at different printing parameters were reported Infill percentage is the parameters that affect the output the most Printing speed has minimal influence on the three responses	[65]
Wang et al. (2021)	PEEK CF/PEEK GF/PEEK	Nozzle temperature, platform temperature, printing speed, layer thickness	Tensile, flexural and impact strengths of PEEK and both PEEK composites were reported Tensile and flexural strengths of all samples increased with increased in both nozzle temperature and platform temperature Higher printing speed and higher layer thickness reduced the mechanical properties	[66]
Amirruddin et al. (2022)	ABS, PLA	Layer thickness, raster angle	A higher layer thickness produces less frictional force and wear Raster angle of 45° produces less friction compared to 0° and 90° ABS has a better wear resistance than PLA.	[67]
Mohd Khairul Nizam et al. (2022)	ABS	Printing orientation	Optimal tensile and impact strengths can be obtained when the sample is printed on edge (YZ) but hardness is the highest when the sample is printed flat (XY)	[68]
Valvez et al. (2022)	PETG PETG/carbon fiber PETG/aramid fiber	Nozzle temperature, speed, layer height, infill	printing parameters optimized for bending strength are slightly different for different material. PETG, nozzle temperature of 265 °C, speed of 20 mm/s, layer height of 0.35 mm and an infill of 100% PETG/carbon fiber, nozzle temperature of 195 °C, speed of 60 mm/s, layer height of 0.53 mm and infill of 100% PETG/aramid fiber nozzle temperature of 265 °C, speed of 20 mm/s, layer height of 0.40 mm and an infill of 100%	[69]
Pang et al. (2022)	PLA	Nozzle temperature	Tensile properties of the PLA specimens increased with printing temperature from 180 °C to 240° C but dimensional accuracy decreased from 180 °C to 240 °C. Optimum temperature for both tensile and dimensional accuracy is 220 °C	[70]
Lokesh et al. (2022)	PLA	Layer height, raster angle, build orientation	Tensile strength decreased with increase in layer height from 0.1 mm to 0.3 mm Maximum tensile strength is observed at 45° build orientation when three build orientations (0°, 45°, 90°) were investigated Raster angle has less impact on mechanical strength	[71]

**Table 3:** The advantages and limitations for synthetic fibers and natural fibers as reinforced material for FDM printed polymer [18,19,106].

Type of Fibers	Advantages	Limitation
Synthetic fibers	Higher strength Higher stiffness Corrosion resistance Flame retardancy Chemical resistance Commercially available	Higher cost Not ‘green’
Natural fibers	Biocompatible, Biodegradable, renewable Recyclable Relatively cheap	Lower strength compared to synthetic fibers Requires treatment of fibers (in general) Fibers are discontinuous (in general) Not all fibers are commercially available

**Table 4:** Brief comparison of fiber reinforced composites, according to length and orientation of fiber [107,111].

Continuous and Aligned Fiber Composites	Discontinuous and Aligned-Fiber Composites	Discontinuous and Randomly Oriented-Fiber Composites
Properties of the composite are highly anisotropic	Properties of the composite are highly anisotropic	Composites are isotropic
Most effective strengthening but only along the designed direction; weaker along other directions	Less effective in strengthening than continuous and aligned fiber composites and only along the designed direction	Least effective in strengthening mechanical but all directions are strengthened
Limited manufacturing methods, hard to be manufactured, the highest cost	Difficult to maintain good alignment of discontinuous fiber during manufacturing; higher cost than discontinuous and randomly oriented-fiber composites	Easier to be manufactured, lowest cost

**Table 5:** Matrix of fiber embedding method and type of fiber length, C—commercially available; R—under research; * Orientation of fibers depends on nozzle width.

	M1 Embedding before the Printing Process	M2 Embedding in the Nozzle	M3 Embedding on the Component
Continuous and aligned fiber composites	Yes, R only	Yes, R only	Yes, R & C
Discontinuous and randomly oriented-fiber composites	Yes *, R & C	No	No
Discontinuous and aligned-fiber composites	Yes *, R & C	No	No

**Table 6:** Selected research of fiber reinforced polymer in FDM by different embedding methods.

Author(s)	Matrix	Reinforcement	Embed. Method	Results	Ref.
Peng et al. (1999)	Epoxy	Fiberglass	M2 (short)	Flexural modulus increased from 4.2 to 6.3 GPa and flexural modulus from 91 MPa to 109 MPa from unaligned to aligned fibers	[90]
Oksman et al. (2003)	PLA, PP	flax	M1 (short)	Results showed that the composite strength of PLA/flax is about 50% better compared to similar PP/flax fiber composites used today in many automotive panels	[143]
Shofner et al. (2003)	ABS	VGCFs, SWCNTs	M1 (short)	UTS = 30 MPa; Young’s Mod = 1.75 GPs; 60% increase in tensile strength over non-reinforced ABS; 68% increase in stiffness	[126]
Masood et al. (2004)	Nylon	Iron	M1 (short)	Tensile modulus = 54 MPa with 30 wt% Iron	[144]
Nikzad et al. (2011)	ABS	Iron, Copper	M1 (short)	Improved stiffness and thermal properties	[93]
Mori et al. (2014)	ABS	CF	M3	Implementing carbon fibers after the nozzle directly into the print job by using ‘dieless forming’ method was proposed. Preliminary results showed strength of composites were improved with addition of carbon fiber	[141]
Tekinalp et al. (2014)	ABS	CF	M1 (short)	The tensile strength and modulus of 3D-printed samples increased ~115% and ~700%	[76]
Ning et al. (2015)	ABS	CF	M1 (short)	Adding carbon fiber into thermoplastic could increase tensile strength and Young’s modulus but may decrease toughness, yield strength and ductility	[41]
Wei et al. (2015)	ABS, PLA	Graphene	M1 (short)	High mechanical strength	[145]
Mahajan et al. (2015)	Epoxy	CF	M2 (short)	Results showed a 44.12% increase in ultimate tensile stress and a 42.67% increase in sample modulus with carbon fiber aligned along the tensile axis	[83]
Matsuzaki et al. (2016)	PLA	CF, Jute	M2 (continuous) Self-modified Blade-1 3D printer	Strength from 40 MPa to 185 MPa; modulus from 4GPa to 20GPa, with decrease in maximum strain	[87]
Li et al. (2016)	PLA	CF	M2 (continuous)	Results indicated that the tensile and flexural strengths of modified carbon fiber reinforced composites were 13.8% and 164% higher than the original carbon fiber reinforced sample	[84]
Tian et al. (2016)	PLA	CF	M2 (continuous) Self-modified printer	Flexural strength of 335 MPa and modulus of 30 GPa were obtained as fiber content reached 27% wt. Nozzle temperature range between 200–230 °C	[80]
Baumann et al. (2017)	ABS	CF GF	M3 Manually	Three different fiber implementation concepts (direct overprint, hypodermic needle, solvent) were used to fabricate polymer composites and found out that direct overprint is the best method among three. They also reported that M3 technique provides a significant increase in tensile strength and elastic modulus for different cases of continuous carbon fiber reinforced polymers	[124]
Thiago et al. (2017)	PLA	CF	M1 (short)	With addition of CF, tensile modulus, and shear modulus of CF+PLA were increased by 2.2 times and 1.16 times	[85]
Nakagawa et al. (2017)	ABS	CF	M3	Carbon fiber was able to reinforce FDM printed ABS Heating (thermal bonding) further improved the strength of the composite	[142]
Ning et al. (2017)	ABS	Chopped CF	M1 (short)	Effects of process parameters such as raster angle, infill speed, nozzle temperature, and layer thickness to the tensile strength of composite were reported	[73]
Eichenhofer et al. (2017)	PLA	PA12/ broken carbon fiber (STS40)	M2	A new manufacture process “continuous lattice fabrication” (CLF) was introduced The new method can increase tensile properties of carbon fiber reinforced PA12 composites, to tensile strength of 560 MPa and elastic moduli of 83 GPa along the fiber direction	[136]
Yang et al. (2017)	ABS	CF	M2 (continuous) Self-developed	Flexural strength of 7127 MPa and flexural modulus of 7.72 GPa; very low interlaminar shear strength of 2.81 MPa	[86]
Dickson et al. (2017)	Nylon	GF, CF, Kevlar fiber	M3	Tensile and flexural behavior of three different composites were compared. Carbon fiber is the best reinforcement for M3 3D printed fiber reinforced Nylon	[81]
Dul et al. (2018)	ABS	GNP, CNT	M1 (short)	Tensile modulus, tensile strength, and creep stability of the nanocomposite, with 6 wt% of GNP, were increased by 47%, 1 % and 42%, respectively, while ABS/CNT nanocomposite showed respective values of 23%, 12% and 20%	[92]
Eichenhofer et al. (2018)	PLA	PA12/ broken carbon fiber (STS40)	M2	Multi-stage pultrusion was able to reduce the void in composite fabricated by CLF processing	[137]
Hu et al. (2018)	PLA	CF	M1 (continuous fiber)	A device was designed to manufacture continuous fiber reinforced thermoplastic (CFRTP) filaments Optimized composite had better flexural strength than the neat PLA	[132]
Liao et al. (2018)	polyamide 12	CF	M1 (short fiber)	Additional of carbon fiber increased the crystallization temperature and degradation temperature. Furthermore, additional of carbon fiber also improved the tensile and flexural strengths, and thermal conductivity	[131]
Chabaud et al. (2019)	PA	CF, GF	M2	Compared to pure PA6. CF/PA and GF/PA have 23 and 19 times higher ultimate tensile strength, respectively, and 137 times higher and 63 times higher for tensile modulus	[146]
Naranjo-Lozada et al. (2019)	Nylon	CF	M3	Continuous fiber reinforced composite fabricated by M3 was compared with nylon sample and Onyx samples (Nylon + carbon fiber, fabricated via M1) Onyn samples had higher elastic modulus and tensile strength than neat Nylon, in all printing intensity or printing patterns Tensile properties of carbon reinforced Nylon increased with the amount of fiber	[46]
Mei et al. (2019)	Nylon	CF	M3	Carbon reinforced Nylon composites were printed at different fiber angles. The sample printed with mixed isotropic fiber angle [0°/45°/90°]^2^ is stronger than samples printed at fiber angles [30°/45°/60°]^2^ and [15°/45°/75°]^2^ Hot pressed composites have higher tensile strength and modulus than the non- hot-pressed composite	[138]
Mohammadizadeh et al. (2019)	Nylon	CF, GF, Kevlar	M3	Tensile, fatigue, and creep behavior of all composites were studied Carbon fiber reinforced composites outperformed GF reinforced composite and Kevlar reinforced composite Failure mechanisms of fiber reinforced Nylon were identified as fiber pull out, fiber breakage, and delamination	[139]
Zhang et al. (2019)	PLA Nylon	Continuous CF Continuous CF	M1 (continuous)	CCF-PLA had higher tensile and bending strength than neat PLA and short carbon fiber reinforced PLA. Similarly, CCF-Nylon had higher bending and tensile strength than neat Nylon	[134]
Bhagia et al. (2020)	PLA	Poplar wood	M1 (short)	Tensile behavior of two poplar-PLA composites (20% milled polar, and 15% fibrillated poplar) were investigated Neat PLA has better tensile behavior than both Poplar wood-PLA composites Variation in tensile strengths of Poplar-PLA composites is due to natural diversity of the poplar wood	[147]
Wang et al. (2020)	PEEK	CF, GF	M1 (short)	Melting point, thermal decomposition temperature and crystallization temperature of both composites are higher than neat PEEK GF/PEEK has better interfacial bonding than CF/PEEK Both composites have better mechanical strengths (tensile, flexural, impact) than neat PEEK Composites with 5 wt.% fiber content are the best in terms of mechanical strength. The increase of fiber content from 5% to 15% reduced the strengths	[125]
Uşun et al. (2021)	PLA	CF	M1 (continuous)	continuous fiber-reinforced thermoplastic (CFRTP) composites with manufactured with continuous fiber-reinforced thermoplastic (CFRTP) filaments The CFRTP filaments were manufactured in house with a melt impregnation line CFRTP composites with 40% CF have higher tensile and flexural strength than composites with 22% CF and 33% CF	[133]
Galos et al. (2021)	Nylon	CF	M3	FDM 3D printed carbon fiber reinforced Nylon has lower longitudinal electrical conductivity than the hot molded composite of similar material. 3D printed composites have better transverse and through -thickness electrical conductivities than the molded composites	[97]
Garofalo et al. (2021)	LDPE Nylon Polycarbonate	CF	M1 (continuous fiber)	A refined manufacturing technique/rig was proposed/built to manufacture continuous fiber reinforced thermoplastic filament The manufactured filament has better prepreg quality and volume fraction but the mechanical properties of 3D printed part by using the new filament were not reported	[135]
Prajapati et al. (2021)	Onyx (Nylon + chopped carbon fiber),	GF	M3	The impact strength of composite was increased with the increment of layer of reinforcement (glass fiber)	[148]
Ahmad et al. (2022)	ABS	Oil palm fiber	M1	Tensile and flexural strengths of composites were optimized through Taguchi experiment. Parameter investigated were layer thickness, printing orientation, Infill density, printing speed. Printing orientation is the most significant printing parameter that affect the tensile and flexural behavior	[149]
Li et al. (2022)	Nylon (PA6)	CF	M1 Custom designed machine	Custom designed screw-extrusion 3D printer was used to produce high strength CF-Nylon composite Addition of carbon fiber reduced composites’ fluidity and porosity	[150]
Man et al. (2022)	Nylon	CF	M3	Scratch behavior of 3D printed CF-PA6 is depended on fiber orientation, fiber distribution and fiber/matrix bonding Abrasion, fiber breakage and fiber removal are the main wear mechanism	[151]
Muller et al. (2022)	PLA	Bamboo Pinewood Cork	M1	Low cycle fatigue of 3D printed PLA and PLA composites were compared. All composites have lower tensile and fatigue behavior compared to neat PLA	[152]

## Data Availability

Not applicable.

## Nomenclature

Additive manufacturing and 3D printing techniques	
3DP	3-Dimensional Printing
AM	Additive Manufacturing
DIW	Direct-Ink-Writing
FDM	Fused Deposition Modelling
FFF	Fused Filament Fabrication
LOM	Laminated Object Manufacturing
SLA	Stereolithography
SLS	Selective Laser Sintering
Polymers	
ABS	Acrylonitrile Butadiene Styrene
HIPS	High-impact Polystyrene
PA	Polyamide
PE	Polyethylene
PEEK	Polyether Ether Ketone
PETG	Polyethylene Terephthalate Glycol
PLA	Poly-Lactic Acid
PP	Polypropylene
PS	Polystyrene
Reinforcement	
CF	Carbon Fiber
GF	Glass Fiber
CNT	Carbon Nanotubes
MWCNT	Multi-walled Carbon Nanotubes
SWCNT	Single-walled Carbon Nanotube
VGCF	Vapor-grown Carbon Fiber
Composites	
FRPC	Fiber Reinforced Polymer Composites
CFRT	Continuous Fiber Reinforced Thermoplastic
